# Integration of sperm ncRNA-directed DNA methylation and DNA methylation-directed histone retention in epigenetic transgenerational inheritance

**DOI:** 10.1186/s13072-020-00378-0

**Published:** 2021-01-12

**Authors:** Daniel Beck, Millissia Ben Maamar, Michael K. Skinner

**Affiliations:** Center for Reproductive Biology, School of Biological Sciences, Washington State University, Pullman, WA 99164-4236 USA

**Keywords:** Epigenetics, ncRNA, DNA methylation, Histone, Transgenerational, Sperm, Non-genetic inheritance, Review

## Abstract

**Background:**

Environmentally induced epigenetic transgenerational inheritance of pathology and phenotypic variation has been demonstrated in all organisms investigated from plants to humans. This non-genetic form of inheritance is mediated through epigenetic alterations in the sperm and/or egg to subsequent generations. Although the combined regulation of differential DNA methylated regions (DMR), non-coding RNA (ncRNA), and differential histone retention (DHR) have been shown to occur, the integration of these different epigenetic processes remains to be elucidated. The current study was designed to examine the integration of the different epigenetic processes.

**Results:**

A rat model of transiently exposed F0 generation gestating females to the agricultural fungicide vinclozolin or pesticide DDT (dichloro-diphenyl-trichloroethane) was used to acquire the sperm from adult males in the subsequent F1 generation offspring, F2 generation grand offspring, and F3 generation great-grand offspring. The F1 generation sperm ncRNA had substantial overlap with the F1, F2 and F3 generation DMRs, suggesting a potential role for RNA-directed DNA methylation. The DMRs also had significant overlap with the DHRs, suggesting potential DNA methylation-directed histone retention. In addition, a high percentage of DMRs induced in the F1 generation sperm were maintained in subsequent generations.

**Conclusions:**

Many of the DMRs, ncRNA, and DHRs were colocalized to the same chromosomal location regions. Observations suggest an integration of DMRs, ncRNA, and DHRs in part involve RNA-directed DNA methylation and DNA methylation-directed histone retention in epigenetic transgenerational inheritance.

## Background

Over the past two decades numerous studies have demonstrated a non-genetic form of inheritance termed epigenetic transgenerational inheritance that is mediated by germline alterations in epigenetic processes [1–3]. One of the first observations involved the environmental agricultural toxicant vinclozolin, which is one of the most commonly used agricultural fungicides, to induce the epigenetic transgenerational inheritance of testis pathology and DNA methylation alterations [1]. Similar observations with a wide variety of environmental toxicants, from dioxin to DDT (dichloro-diphenyl-trichloroethane), have identified similar epigenetic inheritance impacts on a variety of different disease conditions [3–5]. The transgenerational phenotypic manifestations of vinclozolin and DDT include the induction of testis, prostate, kidney, and ovary pathology, as well as obesity [3]. An early observation in mice identified a traumatic stress-induced impact on the epigenetic transgenerational inheritance of behavioral abnormalities [6, 7]. Interestingly, the injection of eggs with the ncRNA from stressed individual male sperm promoted the same transgenerational phenotypes [6]. Subsequent studies have supported a role of either DNA methylation or ncRNA in the germline-mediated epigenetic transgenerational inheritance [3, 8]. This epigenetic transgenerational inheritance phenomenon has been shown to be induced by environmental chemicals, nutrition, stress and trauma abnormalities in rodents and humans [3, 7, 9], as well as a wide variety of environmental stresses in plants [10, 11], insects [12, 13], worms [14], fish [15–17], birds [18, 19], and a variety of mammals such as pigs and humans [20–22]. A number of physiological impacts have been observed including pathologies in the brain, reproductive organs, kidney, immunity, obesity, and infertility [1–3]. The environmentally induced epigenetic transgenerational inheritance phenomenon has been well established, and has significant impacts on disease etiology [2, 3] and other areas of biology such as evolution [23].

Although most previous investigations have focused on an individual epigenetic process such as DNA methylation [3, 4, 10] or ncRNA [6, 8], few have examined multiple processes. Our previous studies demonstrated in both vinclozolin and DDT-induced epigenetic transgenerational inheritance of pathology that the transgenerational F3 generation sperm had coordinately altered differential DNA methylation regions (DMRs), expression of non-coding RNAs (ncRNAs), differential histone retention sites (DHRs), and histone modifications [24, 25]. These observations suggest potential interactions between the different epigenetic processes, but this remains to be elucidated during the epigenetic inheritance phenomenon. Previous studies have demonstrated a role for ncRNA in RNA-directed DNA methylation in a number of different systems [26–28]. The ncRNA can help localize the DNA methylation site and facilitate subsequent chromatin remodeling processes. Therefore, the integration of ncRNA and DNA methylation has been established. Histone modifications can also be modified dramatically by ncRNA and chromatin remodeling in order to transition from euchromatin-active gene expression sites to heterochromatin-inactive sites of DNA [29]. Although information is available on histone retention in sperm and its impacts on the embryo [30, 31], the potential role of different epigenetic processes in histone retention has not been reported. Recently, a role for environmental exposures (e.g., vinclozolin and DDT) to promote transgenerational epigenetic inheritance of sperm histone retention has been observed [24, 25, 32]. The current study investigates the potential integration of DNA methylation, ncRNA, and histone alterations in the epigenetic transgenerational inheritance phenomenon. 

Previous analyses of the concurrent expression of the epigenetic processes between the F1, F2, and F3 generations with a stringent statistical threshold have demonstrated negligible overlap between the different generations or between the epigenetic processes [24, 25]. The current study used an extended overlap analysis with a less stringent statistical threshold and found overlaps between the generations and epigenetic marks. The potential integration of the different epigenetic processes and generational conservation was identified.

## Results

**Fig. 1:**
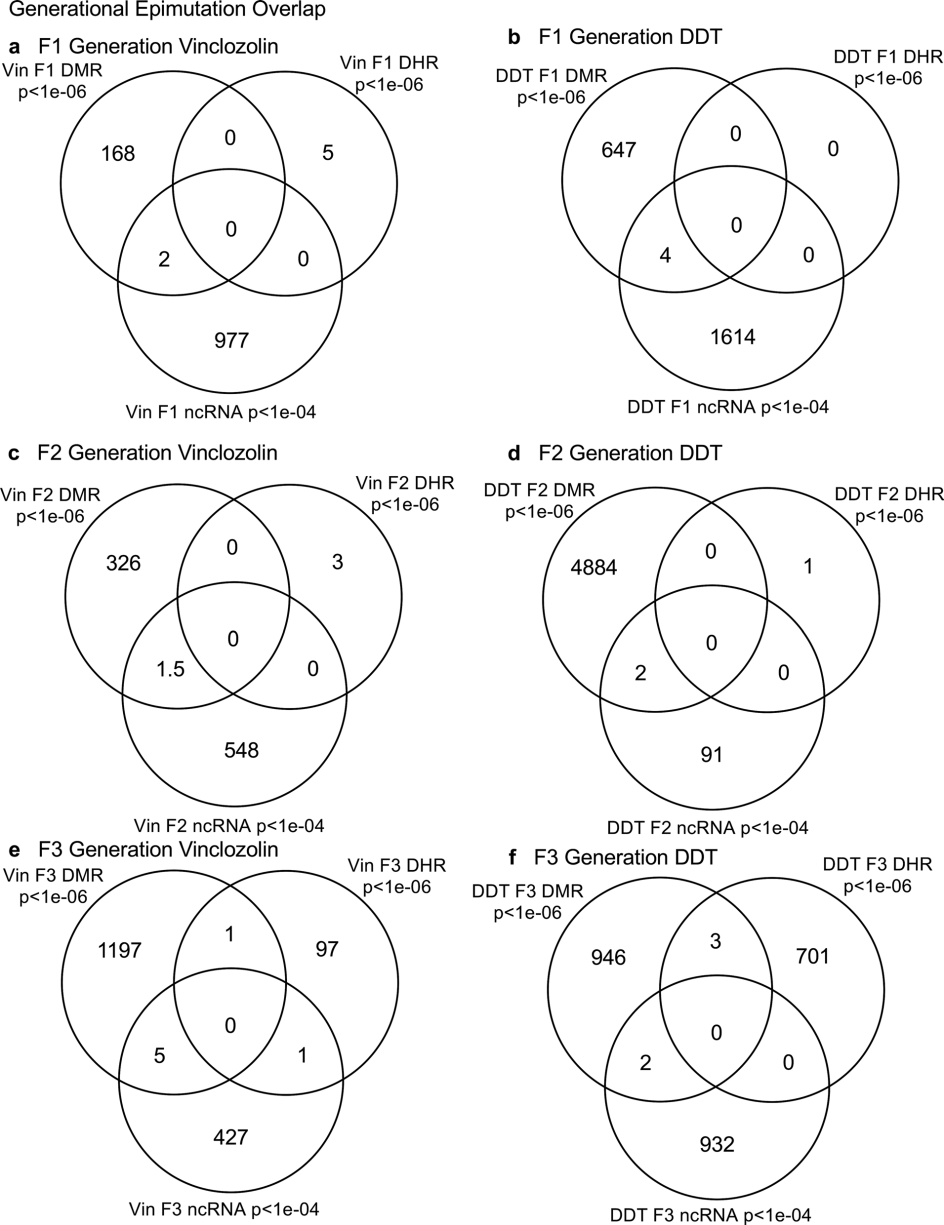
Generational epimutation overlap at high stringent statistical threshold. **a** F1 generation vinclozolin lineage DMR (*p* < 1e−06), DHR (*p* < 1e−06), and ncRNA (*p* < 1e−04). **b** F1 generation DDT lineage DMR (*p* < 1e−06), DHR (*p* < 1e−06), and ncRNA (*p* < 1e−04). **c** F2 generation vinclozolin lineage DMR (*p* < 1e−06), DHR (*p* < 1e−06), and ncRNA (p < 1e−04). **d** F2 generation DDT lineage DMR (*p* < 1e−06), DHR (*p* < 1e−06), and ncRNA (*p* < 1e−04). **e** F3 generation vinclozolin lineage DMR (*p* < 1e−06), DHR (*p* < 1e−06), and ncRNA (*p* < 1e−04). **f** F3 generation DDT lineage DMR (*p* < 1e−06), DHR (*p* < 1e−06), and ncRNA (*p* < 1e−04)

The experimental design involved F0 generation gestating outbred Sprague Dawley female rats at 120 days of age being exposed during embryonic days 8–14 (E8–E14) transiently to vinclozolin (100 mg/kg body weight/day), or DDT (25 mg/kg body weight/day), or vehicle dimethyl sulfoxide (DMSO) control, as previously described [24, 25]. The F1 generation offspring were obtained and aged to 90 days of age then bred within the lineage (control, vinclozolin, or DDT) in order to generate the F2 generation grand offspring. Afterward, the F2 generation was similarly bred to generate the transgenerational F3 generation great-grand offspring within the lineage. At each generation or lineage no sibling or cousin breeding was used to avoid any inbreeding artifacts [1, 3]. Litter bias was avoided by culling litters to 10 (approximately 5 females and 5 males), and then only one or two males and females from each litter being used for breeding within the lineage, as previously described. All males were aged to 120 days and sacrificed for sperm collection for molecular analysis, as described for previous reported studies [24, 25]. The number of individual animals investigated at each generation for sperm collection and molecular analysis was approximately 10–17 males, so *n* = 10–17 for animals with three different pools of 4–6 animals for each generation and epimutation analysis. The sperm collected were used to isolate RNA, DNA, and chromatin for analysis of ncRNA, DNA methylation, histone retention, and histone modification, as described in previous studies [24, 25], (Fig. 1). The molecular data from these previous studies (GEO # GSE109775, GSE106125, and NCIB SRA: PRJNA430483 largeRNA (control and DTT), PRJNA430740 smallRNA) were analyzed to explore data further bioinformatically.

The sperm DMRs, ncRNA (both small sncRNA and large lncRNA), and DHRs were analyzed in each sample, as previously described [24, 25], for the vinclozolin and DDT F1, F2 and F3 generation sperm samples. The numbers and overlaps of DMRs, ncRNA, and DHRs for each generation with a high stringency threshold are presented as previously reported in (Fig. 1). The overlaps with a Venn diagram for the transgenerational F3 generation for the different epigenetic marks is negligible with the high stringency threshold, (Fig. 1e, f), for each exposure, as previously identified [24, 25]. The F1 and F2 generations also were primarily distinct among the epimutations, (Fig. 1a–d). Although the different epigenetic alterations are present at each generation for both exposure lineages, the overlaps with a stringent statistical threshold were negligible, suggesting distinct functions and a lack of integration, as previously suggested [24, 25].

**Fig. 2:**
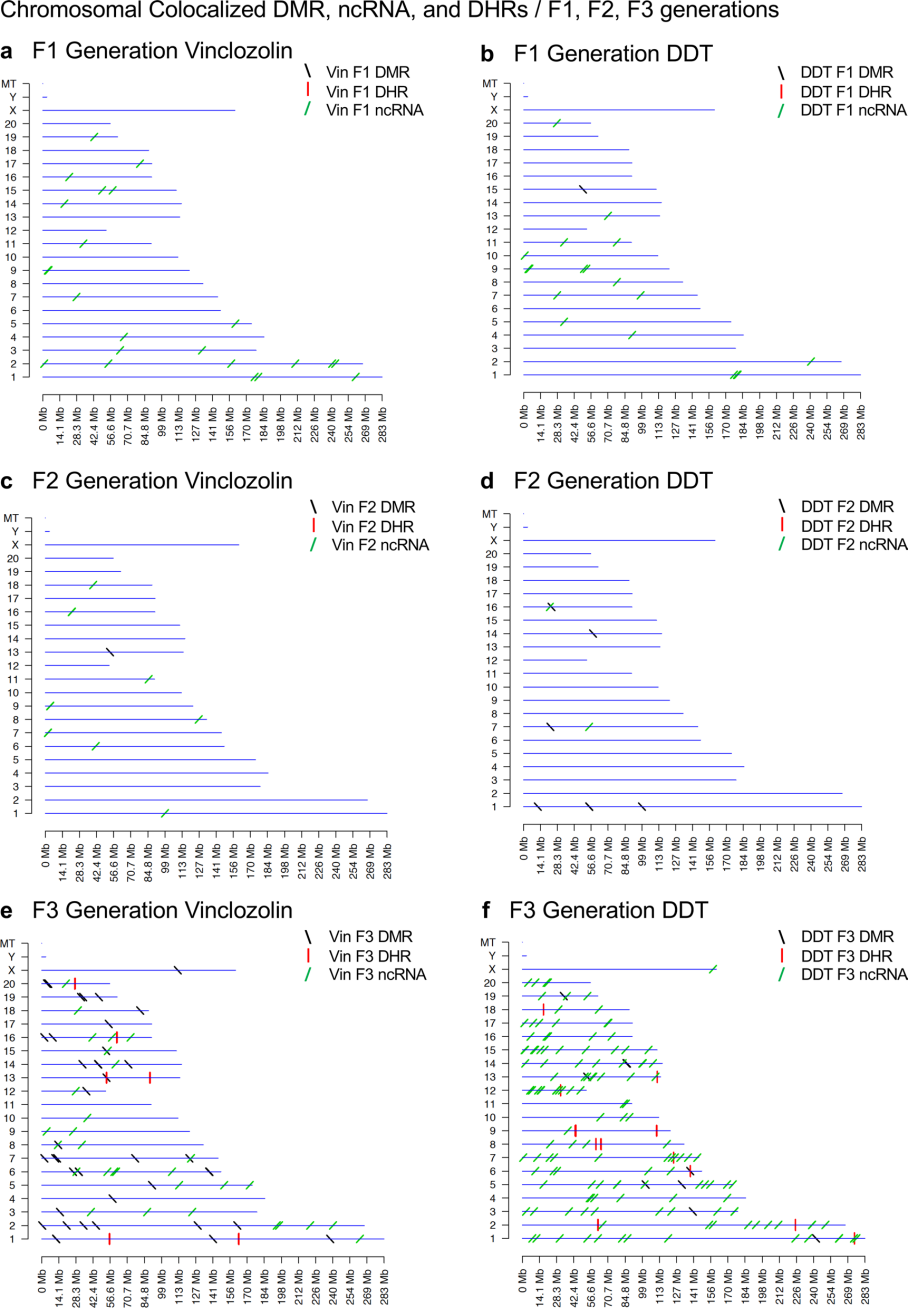
Chromosomal colocalization of overlap epimutations. The overlap of one epimutation at high statistical stringency (DMR *p* < 1e−06, DHR *p* < 1e−06, or ncRNA *p* < 1e−04) overlap with others at *p* < 0.05. The epimutation at high stringency is identified with color and marked as indicated by the inset legend. The chromosomal number and size (megabase) are presented. **a** F1 generation vinclozolin lineage ncRNA and DMR. **b** F1 generation DDT lineage ncRNA and DMR. **c** F2 generation vinclozolin lineage ncRNA and DMR. **d** F2 generation DDT lineage ncRNA and DMR. **e** F3 generation vinclozolin lineage DMR, DHR and ncRNA. **f** F3 generation DDT lineage DMR, DHR and ncRNA

Interestingly, when a comparison of one epimutation at a high stringency was made to the others at *p* < 0.05, a number of genomic locations were identified with the different types of epimutations present. The chromosomal locations of these altered epigenetic marks (i.e., epimutations) are presented in (Fig. 2) and in (Additional file 1: Tables S1–S6) for each generation for both vinclozolin and DDT lineage sperm samples. The color-coded labels identify the DMR, ncRNA, and DHRs throughout the genomes with common chromosomal locations for each generation. Only those sites significant at high stringency (color code index) with one epimutation analysis that overlap with the other epimutations at *p* < 0.05 are shown, (Fig. 2). Specific epimutation chromosomal locations, statistical p-values, and gene associations are presented in (Additional file 1: Tables S1–S6). In the F1 and F2 generations only the alterations in ncRNAs and DMRs were found, as previously described [24, 25]. Therefore, the overlaps were primarily between the ncRNA and DMR in the F1 and F2 generations, (Fig. 2a–d). The DHRs developed in the transgenerational F3 generation, as previously described [24, 25]. In the F1 and F2 generations the ncRNA was predominantly the high statistically significant epimutation and overlap with DMR at the *p* < 0.05, (Fig. 2a–c), with a mix of ncRNA and DMR in the DDT lineage F2 generation, (Fig. 2d). The transgenerational F3 generation also had a mix of ncRNA and DMR at a high statistical significance, as well as a number of DHR, (Fig. 2e, f). Therefore, chromosomal locations with multiple epimutations are identified with ncRNA being predominant in the F1 and F2 generations with the high statistical threshold, and DMRs being more predominant in the F3 generation with a mix of the various epimutations, (Fig. 2 and Additional file 1: Tables S1–S6).

**Fig. 3:**
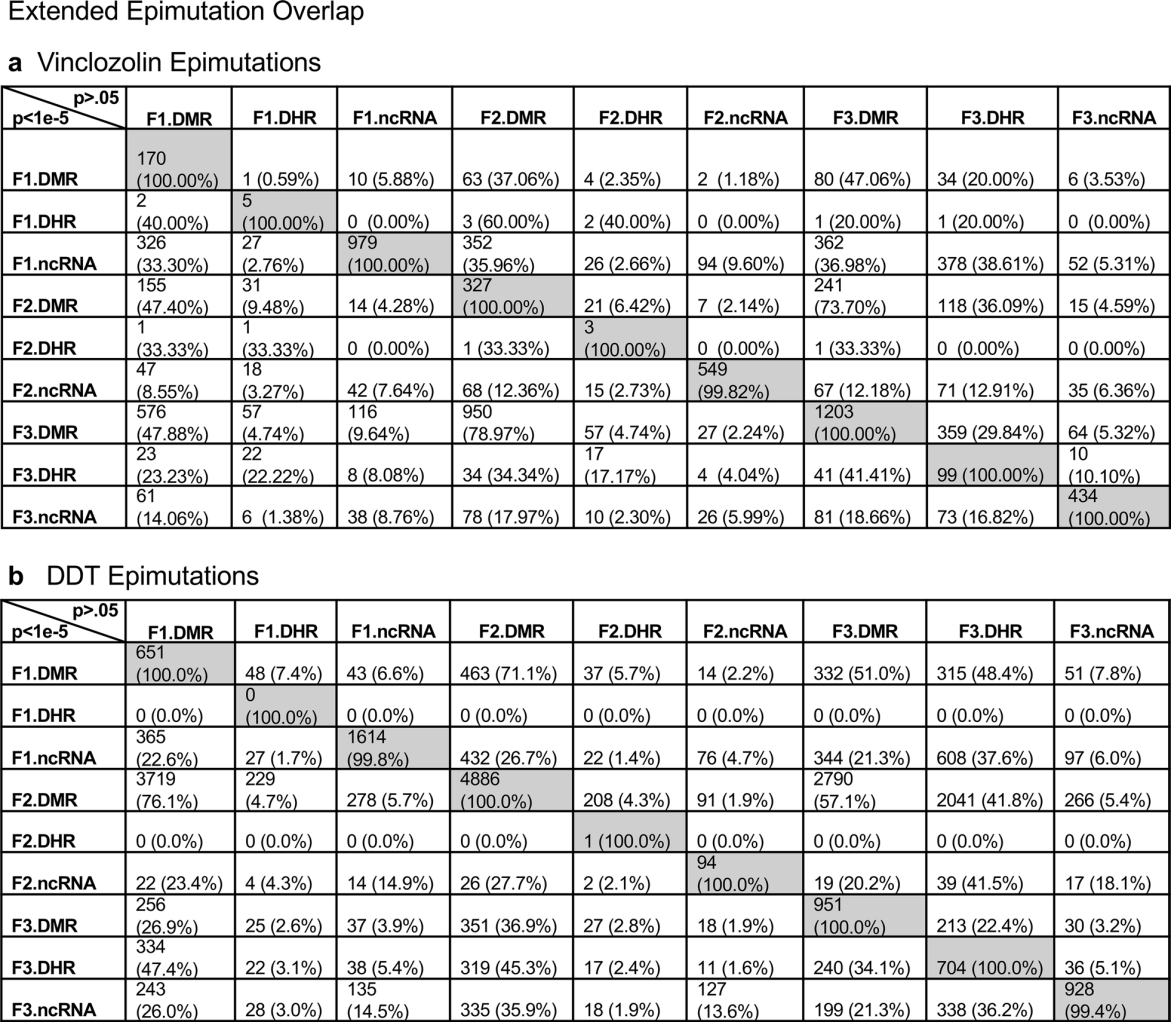
Extended epimutation overlap. The epimutations at high stringency (DMR *p* < 1e−06, DHR *p* < 1e−06, and ncRNA *p* < 1e−04) in rows were compared to epimutations at *p* < 0.05 in columns. The number of overlap epimutations and percentage of the total are presented for each overlap. As anticipated, 100% overlap was observed for the same generation and epimutation indicated by shaded box. **a** Vinclozolin lineage epimutation and **b** DDT lineage epimutation overlap

**Fig. 4:**
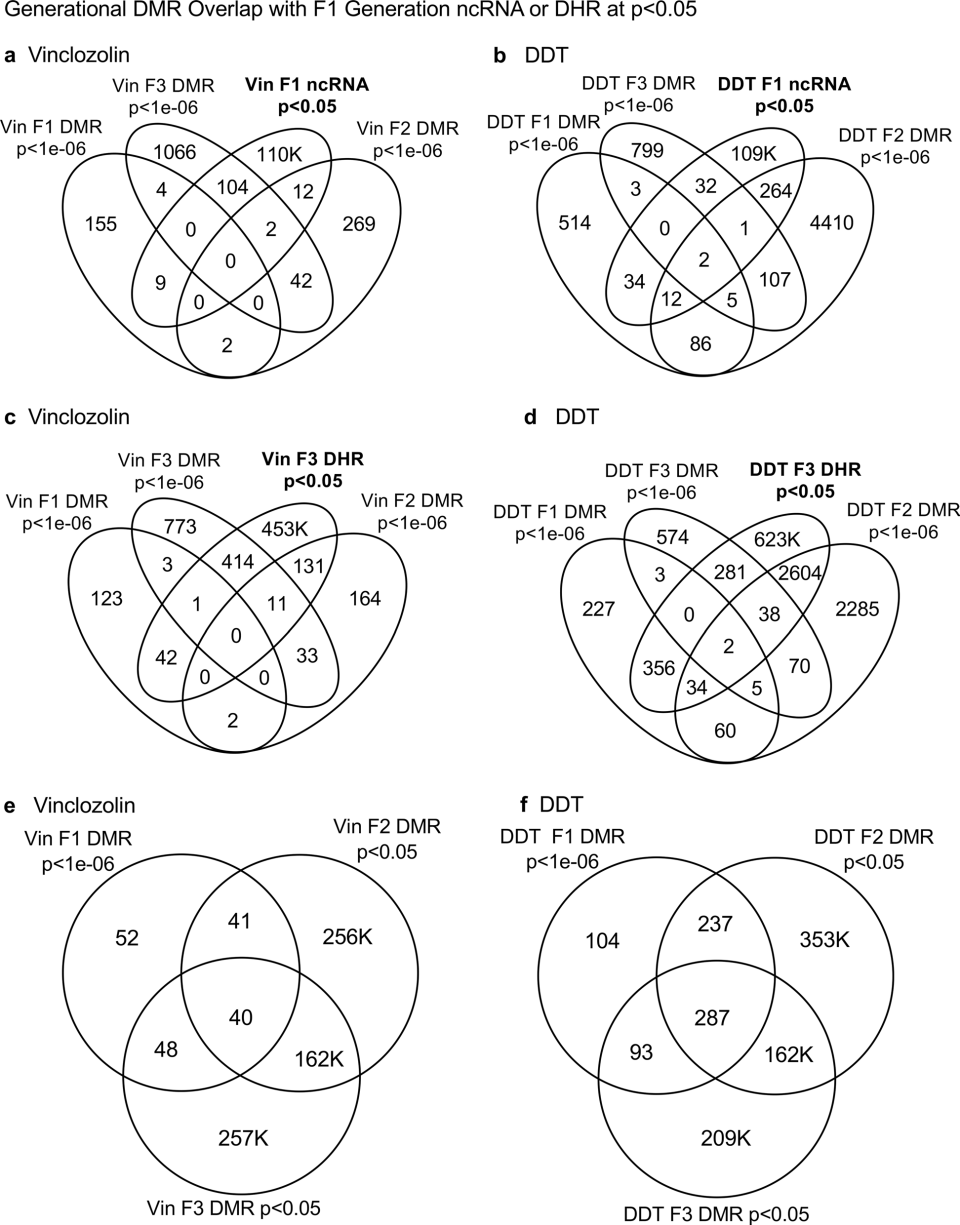
Epimutation overlaps. Generational DMR overlap with F1 generation ncRNA *p* < 0.05. A Venn diagram overlap of F1, F2, and F3 generation DMR (*p* < 1e−06) with F1 generation ncRNA (*p* < 0.05). **a** Vinclozolin lineage DMR and ncRNA overlap. **b** DDT lineage DMR and ncRNA overlap. Generational DMR overlap with F3 generation DHR *p* < 0.05. A Venn diagram overlap of F1, F2, and F3 generation DMR (*p* < 1e−06) with F3 generation DHR (*p* < 0.05). **c** Vinclozolin lineage DMR and DHR overlap. **d** DDT lineage DMR and DHR overlap. Generational DMR overlap. A Venn diagram overlap of F1 generation DMR (*p* < 1e−06) with F2 and F3 generation DMR (*p* < 0.05). **e** Vinclozolin lineage DMR overlap. **f** DDT lineage DMR overlap

An extended overlap analysis was performed with both the DDT and vinclozolin lineage data using a less stringent statistical threshold for the comparisons, (Fig. 3). The more stringent statistical threshold epigenetic data sets (DMRs *p* < 1e−06, ncRNA *p* < 1e−04, and DHRs *p* < 1e−06) were compared between the generations and the epigenetic marks with a *p* < 0.05 statistical threshold. This optimized the potential to identify overlaps compared to the more stringent thresholds used in (Fig. 1). The rows present the more stringent threshold DMRs, ncRNA, and DHRs for the F1, F2, and F3 generations. The columns present the corresponding *p* < 0.05 threshold overlaps with the higher p-value threshold data sets. Examination of the horizontal rows, as expected, show 100% overlap (i.e., shaded) for the same data set and the number of associated epigenetic marks and percentage (%) overlap with the left margin value. This extended overlap allows the two different threshold stringencies to be compared and to determine additional overlap observations, (Fig. 3). Similar trends in the overlaps are observed for both the DDT and vinclozolin data sets. One of the initial observations was that the F1 generation ncRNA had a high percentage overlap with the F3 generation DMR, (Fig. 3 and Additional file 1: Tables S1 and S2). Similar observations are made with the F1 and F2 generations. For the F1 generation, DDT ncRNA had over a 20% overlap observed with the F1, F2, and F3 generation DMRs, while vinclozolin F1 generation ncRNA had approximately 35% overlap with the F1, F2, and F3 generation DMRs, (Figs. 3 and 4a, b). The lists of overlapping ncRNA and DMR sites are presented in (Additional file 1: Tables S1 and S2). The F2 and F3 generation ncRNA were similar with the overlap with the DDT generation DMRs of approximately 20%, but reduced to 10–15% with the vinclozolin DMRs, (Fig. 3). Therefore, some ncRNAs were common between the generations, and had overlap with the DMRs that ranged between 8–35% overlap for the vinclozolin DMRs and 15–20% overlap for DDT DMRs. The potential that the ncRNA may promote RNA-directed DNA methylation is suggested. The Venn diagrams presented in (Fig. 4a, b) support those overlaps and the epigenetic ncRNA and DMR overlaps are listed in (Additional file 1: Tables S1 and S2).

The next observation was that the F1, F2, and F3 generation DMRs had a 20–48% overlap with the F3 generation DHRs for the DDT and vinclozolin lineages, (Fig. 3). Interestingly, the F3 generation DHRs had a 23–47% overlap with the F1, F2, and F3 generation DMRs for both exposure lineages. The Venn diagram overlaps in (Fig. 4c, d) support these DMR and DHR overlaps and suggests DMRs may help guide DHR formation transgenerationally. The overlapping F3 generation DMRs and DHRs are presented in (Additional file 1: Tables S3 and S4).

An interesting observation was the overlap between the F1, F2, and F3 generation DMRs for both DDT and vinclozolin exposures, (Figs. 3 and 4e, f). The highest overlap for the DDT F1 generation DMRs was the F2 generation DMRs with a 71% overlap, and for the vinclozolin F2 generation DMRs with the F3 generation DMRs with a 73% overlap. The highest for the F3 generation DMRs was 79% overlap with the vinclozolin F2 DMRs. Generally, a 25–50% overlap existed between the F1, F2, and F3 generation DMRs for both exposures, (Fig. 3). A Venn diagram supports this observation and demonstrates approximately 25% overlap for the vinclozolin DMRs and 35% overlap for the DDT DMRs, (Fig. 4e, f). Lists of these overlapping DMRs are presented in (Additional file 1: Tables S5 and S6). Therefore, a percentage (25–35%) of the F1 generation individual DMRs were retained transgenerationally.

**Fig. 5:**
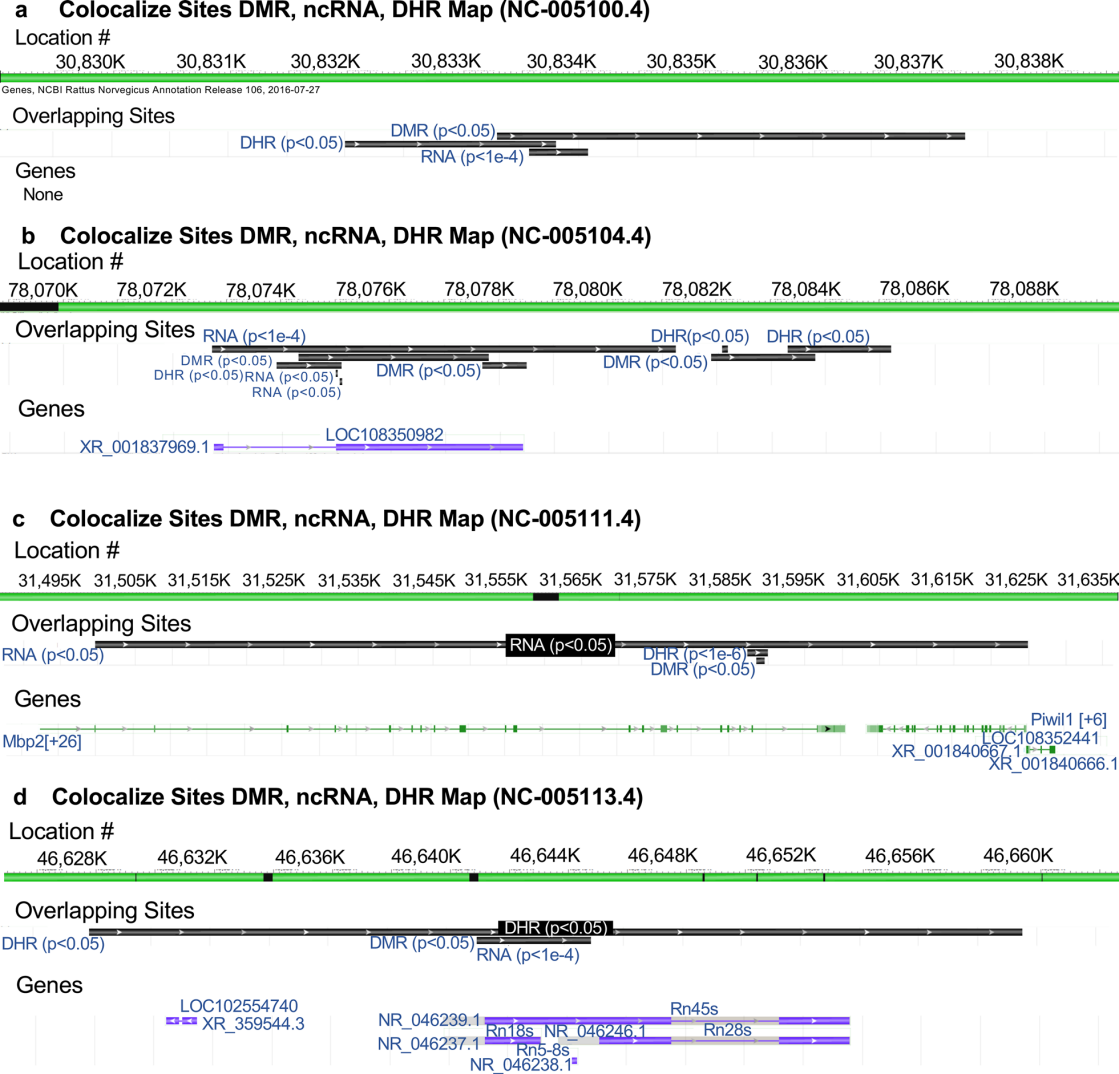
Genomic colocalization of DMR, DHR and ncRNA. The genomic and colocalized DMR, DHR and ncRNA presented. The region size (bp), genes present, and localization of DMR, DHR and ncRNA identified. The various examples include **a** nc-005100.4, **b** nc-005104.4, **c** nc-005111.4, and **d** nc-005113.4 from the NCBI Rattus norvegicus release 106 in 2016

Generally, the F3 generation epigenetic alterations had more overlap among each other and with the other generations for both exposures. A Venn diagram analysis was used to identify the epigenetic sites with overlapping DMRs, ncRNA, and DHRs, (Fig. 4). The overlapping F3 generation epigenetic sites were approximately 25% for vinclozolin and DDT lineages. A permutation analysis was performed to demonstrate this is significantly greater than the random overlap observed, with a *p* value of *p* ≤ 0.05 for both 1 kb and 10 kb overlapping sites. Several sites were randomly selected and are mapped to identify the overlapping chromosomal locations of the DMR, ncRNA, and DHR, (Fig. 5). The actual statistical significance of the overlapping epimutations in these examples includes: (Fig. 5a) (ncRNA *p* < 1e−04, DHR *p* < 0.03, and DMR *p* < 0.005); (Fig. 5b) (ncRNA *p* < 1e−04, DHR *p* < 0.001, and DMR *p* < 0.0004); (Fig. 5c) (DHR *p* < 1e−08, ncRNA *p* < 0.005, and DMR *p* < 0.04); and (Fig. 5d) (ncRNA *p* < 1e−06, DMR *p* < 1e−04, and DHR *p* < 1e−05). The potential that RNA-directed DNA methylation and DMR-directed histone retention is involved is reviewed in the “Discussion” section.

**Fig. 6:**
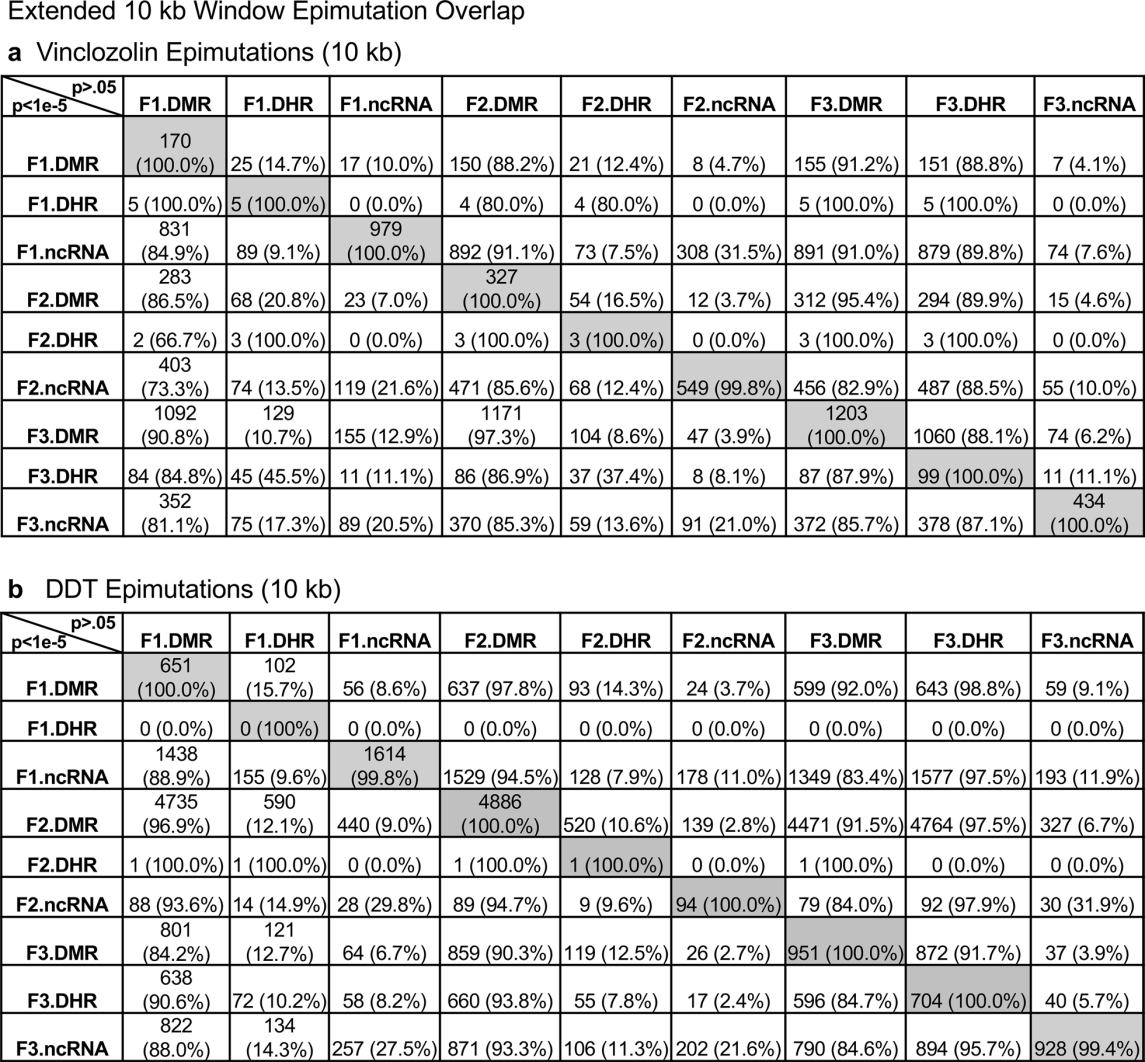
Extended epimutation overlap within a 10-kb region. The epimutations at high stringency (DMR *p* < 1e−06, DHR *p* < 1e−06, and ncRNA *p* < 1e−04) in rows were compared to epimutations at *p* < 0.05 in columns. The number of overlap epimutations and percentage of the total are presented for each overlap. As anticipated, 100% overlap was observed for the same generation and epimutation indicated by shaded box. **a** Vinclozolin lineage epimutation and **b** DDT lineage epimutation overlap

All the previous analyses and overlaps presented were based on a direct overlapping chromosomal location for the ncRNA, DMR and DHR. The question was addressed if a greater number of sites exist with epimutations that were in the same region but not directly overlapped. A 5 kb distance on either side of the epimutations was used to have a 10 kb window for the potential overlapping region. An extended overlap using this 10 kb window was used with the same data that will identify sites that directly overlap and those nearby within the 10 kb window, Fig. 6. The level of overlap with a 10 kb window identified the same overlaps presented and discussed, but the level of overlap was in the 80–99% range, (Fig. 6). Both the vinclozolin and DDT lineage had the same high level of overlap with most being > 90% range, with a permutation analysis *p* value of *p* < 0.001. Using this 10 kb window the majority of ncRNA, DMRs and DHRs overlapped between the generations and epimutations. This supported all the previous observations and demonstrated a significant level of epimutation overlap. Since approximately 90% of the F3 generation DMRs overlapped with the F3 generation DHRs and F1 generation ncRNA, the conserved F3 generation DMRs, (Additional file 1: Table S5 and S6), were used in a Pathway Studio analysis to link DMR associated genes with cellular processes and pathologies, (Additional file 1: S1 and S2). A large number of the DMR and epimutation associated genes linked to various transgenerational pathologies previously observed, including kidney disease, mammary tumors, immune abnormalities, prostate disease, metabolic disease, or behavioral abnormalities [3, 33, 34].

## Discussion

Previous studies have demonstrated the concurrent presence of DMRs, ncRNA, and DHRs in sperm following DDT or vinclozolin exposure of F0 generation gestating females during gonadal sex determination [24, 25]. These data were obtained and reported at a stringent statistical threshold selection and demonstrated negligible overlap at each generation, (Fig. 1) [24, 25]. The current study was designed to further investigate the potential integration of the different epigenetic processes between the F1, F2, and F3 generations. An approach was taken to compare the more stringent statistical threshold values for DMRs, ncRNAs, and DHRs with the less stringent *p* < 0.05 threshold between the different epigenetic processes and generations. This extended overlap approach generated a number of observations to suggest an integration between generations for the epigenetic transgenerational inheritance phenomenon.

An interesting observation from the extended overlap of DMRs demonstrated that approximately 40–50% of the F1 generation sperm DMRs were retained and also present in the F2 and transgenerational F3 generations, (Figs. 3 and 4e, f). This was 88–97% of the DMRs when 10 kb windows were used, (Fig. 6). A permutation analysis demonstrated this was significant (*p* < 0.001) and not due to random associations. The list of conserved F1 generation DMRs in subsequent generations is presented in (Additional file 1: Tables S5 and S6, and those DMRs with gene associations suggest approximately 50% of these conserved DMRs were associated with genes. Many of these genes had associations with a variety of pathologies, Additional file 1: Figures S1 and S2). Therefore, a percentage of the F1 generation sperm DMRs were programmed and then conserved in subsequent generations. Although a majority of the F1 generation sperm DMRs were conserved generationally, there were minimal similarities between the different generations for ncRNAs, (Figs. 3 and 6). The DHRs were primarily present in the F3 generation sperm, so not conservation between generations, (Fig. 3. ) In contrast, when a 10 kb region is considered approximately 40% of the F3 generation DHRs are present in the F1 and F2 generations, (Fig. 6). The potential role of these DMRs for guided transgenerational histone retention is discussed below.

**Fig. 7:**
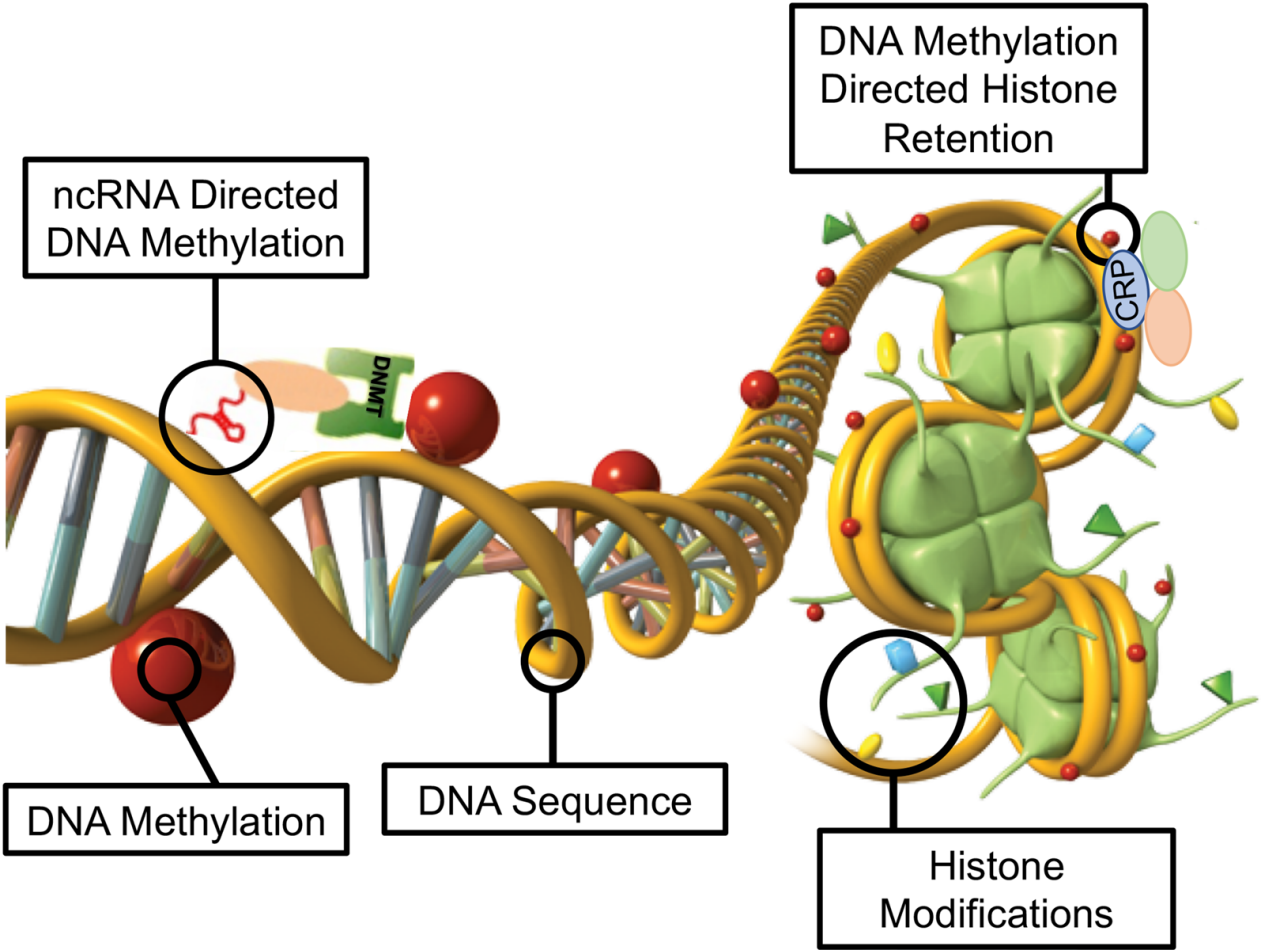
Diagram of ncRNA-directed DNA methylation and DNA methylation-directed histone retention. The red dot identifies DNA methylation, green histone the nucleosome with modifications in histone tails indicated. The ncRNA association with cofactors and DNA methyltransferase (DNMT) promoting DNA methylation (red dot) for RNA-directed DNA methylation. The DNA methylation (red dot) association with chromatin remodeling proteins (CRP) to promote histone retention is indicated

The second interesting observation was the overlap of the F1 generation sperm ncRNA with the F1, F2, and F3 generation DMRs. Over 20% in DDT and 35% in vinclozolin F1 generation ncRNA overlapped with the F1, F2, and F3 generation DMRs, (Figs. 3 and 4 and Additional file 1: Tables S1 and S2). Observations suggest the potential role of ncRNA-directed DNA methylation in the direct exposure F1 generation and transgenerational F3 generation. Previous literature has established a role for RNA-directed DNA methylation in a number of biological and cellular systems [26–28]. This involves the ability of the ncRNA to recruit or direct chromatin remodeling proteins and proteins such as DNA methyltransferase to guide the DNA methylation at a chromosomal site, which has been established in a variety of different organisms and developmental processes [26–28]. Observations suggest ncRNA-directed DNA methylation may have a role in the epigenetic transgenerational inheritance phenomenon. Although the F1 generation ncRNA have the highest overlap with the F3 generation DMRs, overlaps are also observed with the F1 and F2 generation ncRNA with the various generation DMRs, (Fig. 3). When a 10 kb region overlap is considered, the F1 generation ncRNAs have a 91% overlap with the F2 and F3 generation DMRs, (Fig. 6). The overlaps of the ncRNA and DMRs suggest ncRNA-directed DNA methylation has a potential role in the epigenetic transgenerational inheritance process, (Fig. 7). A combination of F1 generation direct exposure alterations in ncRNA and subsequent transgenerational F3 generation actions on DNA methylation appears to be involved. The colocalized epigenetic sites with ncRNA and DNA methylation support this proposal, (Fig. 5). Although the molecular process of RNA-directed DNA methylation has been established [26–28], and suggested in generational impacts in plants and humans [35, 36], the current study only demonstrates the strong correlations of the ncRNA and DMRs. Future studies are needed to provide more molecular insights and validation of the ncRNA-directed DNA methylation in the epigenetic transgenerational phenomenon.

Another interesting observation was the overlap of the transgenerational F3 generation DMRs with the DHRs. Although negligible DHRs are present in the F1 or F2 generations, the F3 generation has DHRs that overlap with F1 and F2 generation DMRs, (Fig. 3). For the DDT DMRs there was a range of 35–50% overlap and for vinclozolin DMRs, a 23–41% overlap. Considering a 10 kb region overlap, the F3 generation DHRs had an 85–95% overlap with the DMRs at all the generations, (Fig. 6). The permutation analysis demonstrated this number of 10 kb region overlaps is not due to random associations (*p* < 0.001). The literature for spermatid exchange of histones for protamines to condense the DNA into the head of the sperm is well established in most organisms investigated [37–39]. Although the vast majority of the sperm DNA has associated protamines, a percentage of the histones are retained, which varies between 5–10% of the DNA in different mammalian species [40]. Previously, we found histone retention was significantly increased in the transgenerational F3 generation sperm with the presence of new retention sites [24, 25, 32]. Therefore, an additional epigenetic mechanism influenced during the epigenetic transgenerational inheritance process involves altered histone retention [32]. Previous literature has described the transition proteins and processes of the replacement of histones for protamines [41, 42], but the role of epigenetic processes such as DNA methylation have not been considered. Our previous observations suggest a role for this process in epigenetic inheritance [24, 25]. The current study indicates a potential role for DNA methylation in guiding or directing histone retention,(Figs. 3 and 6). Previous studies have demonstrated a critical role for DNA methylation in the actions of chromatin remodeling proteins [41–43]. So, DNA methylation could alter the associated proteins and secondary structure of DNA that is an aspect of the process of histone retention. Although further investigation of the molecular processes is required in future studies, the observations from the current study suggest a potential role of DNA methylation-directed differential histone retention, (Fig. 7). The DMRs are proposed to assist in the guiding or directing of histone retention sites such that an increased number of sites appear transgenerationally. Therefore, the existence of DNA methylation-directed histone retention is proposed, and the observations support an integration of DMRs and DHRs transgenerationally. An interesting additional observation is the F1 and F2 generation DMRs that develop following direct exposure to toxicants are similar to the F3 generation DMRs, but that the DHRs did not form until the transgenerational F3 generation, (Figs. 3 and 6).

The current study findings help integrate the previous data obtained with ncRNA, DMRs, and DHRs [24, 25]. Potential roles of ncRNA-directed DMRs and DMR-directed DHRs are suggested. A percentage of the F1 generation DMRs are retained and conserved for subsequent F2 and F3 generations. The F1 generation ncRNA overlapped with the F2 and F3 generation DMRs, supporting the role for ncRNA-directed DNA methylation and formation of DMRs, (Fig. 7). The specific subtypes of sncRNA and lncRNA in this process will require further investigation. The potential for DMR-directed DHRs is suggested, but further information is required to elucidate the specific processes involved. Approximately half of the overlapping epimutations had associated known genes. Many of these genes are associated with previously identified pathologies, (Additional file 1: Figures S1 and S2), so support a mechanism for transgenerational pathology. The proposed model and integration of the transgenerational ncRNAs, DMRs and DHRs are presented in (Fig. 7). The current study observations suggest the integration of epigenetic processes in the epigenetic transgenerational phenomenon. Insights are provided into the development and generational transmission of these environmentally induced sperm epimutations that have previously been shown to associate with disease development and etiology. The potential use of these integrated epigenetic chromosomal sites as biomarkers to identify exposure and/or disease susceptibility suggests they could be used as diagnostics to facilitate preventative medicine in the future. Further investigation is needed to more thoroughly establish these mechanisms in the epigenetic transgenerational inheritance phenomenon, but the current study provides support and a framework for the integration of the various epigenetic processes.

## Conclusions

The observations with the two different exposures of DDT or vinclozolin suggest the generational impacts and transgenerational integration of the ncRNA, DMRs, and DHRs are similar. Variation in the percent overlaps is observed, but the same trends and conclusions of integration of the various epimutations are similar for both DDT and vinclozolin exposure lineages. The colocalized epimutation sites for the different exposures demonstrate the same phenomenon, but independent sites are observed for each exposure. The two different models of environmentally induced epigenetic transgenerational inheritance support the general mechanism proposed for ncRNA-directed DNA methylation and DMR-directed DHR development. Although the current study identifies such colocalized and interacting epimutation sites, many of the specific ncRNAs, DMRs and DHRs are not colocalized [24, 25]. Therefore, independent actions of ncRNAs, DMRs and DHRs will also be important in the mechanism involved in environmentally induced epigenetic transgenerational inheritance. A combination of ncRNA, DMR, and DHR epimutations developed during gametogenesis allows for post fertilization embryonic impacts and suggests integration of ncRNA and DMR will be involved in the epigenetic inheritance. The proposed mechanism in (Fig. 7) helps elucidate the molecular mechanisms involved in the epigenetic transgenerational inheritance phenomenon.

## Materials and methods summary

### Animal studies and breeding

As previously described [24, 25] and expanded in the (Additional file 1: Supplemental Methods), outbred Sprague Dawley SD male and female rats were fed a standard diet with water ad lib and mated. Gestating female rats were exposed to DDT or vinclozolin, and offspring were bred within each lineage for three generations in the absence of exposure. The F3 generation was aged to 120 days for sperm isolation and molecular analysis, as described in the (Additional file 1: Supplemental Methods). Sperm were isolated and used for epigenetic analysis, as described in the (Additional file 1: Supplemental Methods). All experimental protocols for the procedures with rats were pre-approved by the Washington State University Animal Care and Use Committee (protocol IACUC # 6252), and all methods were performed in accordance with the relevant guidelines and regulations.

### Epigenetic analysis, statistics and bioinformatics

As previously described [44], DNA was isolated from sperm collected at the time of dissection. The DNA isolation protocol has been previously described [33, 34], (Additional file 1: Supplemental Methods). Methylated DNA immunoprecipitation (MeDIP), followed by next generation sequencing (MeDIP-Seq) was performed on the isolated DNA. MeDIP-Seq, sequencing libraries, next generation sequencing, and bioinformatics analysis were performed, as described previously [33, 34] and in the (Additional file 1: Supplemental Methods). All molecular data has been deposited into the public database at NCBI (GEO # GSE109775 and GSE106125), and R code computational tools are available at GitHub (https://github.com/skinnerlab/MeDIP-seq) and https://skinner.wsu.edu/genomic-data-and-r-code-files/.

## Supplementary Information

**Additional file 1:** **Figure S1.** Vinclozolin lineage F3 generation conserved DMR in common with DHR and F1 generation ncRNA. **Figure S2.** DDT lineage F3 generation conserved DMR in common with DHR and F1 generation ncRNA. **Table S1.** Vinclozolin lineage F1 generation ncRNA & F1, F2 and F3 generation DMR overlap list. **Table S2.** DDT lineage F1 generation ncRNA & F1, F2 and F3 generation DMR overlap list. **Table S3.** F3 generation vinclozolin DMR & F3 generation DHR overlap list. **Table S4.** F3 generation DDT DMR & F3 generation DHR overlap list.** Table S5.** Vinclozolin lineage F1, F2, F3 generation DMR overlap list. **Table S6.** DDT lineage F1, F2, F3 generation DMR overlap list.

## Data Availability

All molecular data have been deposited into the public database at NCBI (GEO # GSE109775 and GSE106125, NCIB SRA accession numbers: PRJNA430483 largeRNA (control and DTT), PRJNA430740 smallRNA (control, vinclozolin and DTT)). The specific scripts used to perform the analysis can be accessed at github.com/skinnerlab and at www.skinner.wsu.edu/genomic-data-and-r-code-files.
