# Supplementary material for: Integration of sperm ncRNA-directed DNA methylation and DNA methylation-directed histone retention in epigenetic transgenerational inheritance
Source: Epigenetics Chromatin. 2021 Jan 12;14:6. doi: 10.1186/s13072-020-00378-0 (PMC7802319; doi:10.1186/s13072-020-00378-0)
Supplement: Supplementary file 1 — Additional file 1: Figure S1. Vinclozolin lineage F3 generation conserved DMR in common with DHR and F1 generation ncRNA. Figure S2. DDT lineage F3 generation conserved DMR in common with DHR and F1 generation ncRNA. Table S1. Vinclozolin lineage F1 generation ncRNA & F1, F2 and F3 generation DMR overlap list. Table S2. DDT lineage F1 generation ncRNA & F1, F2 and F3 generation DMR overlap list. Table S3. F3 generation vinclozolin DMR & F3 generation DHR overlap list. Table S4. F3 generation DDT DMR & F3 generation DHR overlap list. Table S5. Vinclozolin lineage F1, F2, F3 generation DMR overlap list. Table S6. DDT lineage F1, F2, F3 generation DMR overlap list. [file 13072_2020_378_MOESM1_ESM.pdf]

Supplemental Methods

Animal studies and breeding

As previously described (1, 2), female and male rats of an outbred strain Hsd:Sprague Dawley SD (Harlan) at about 70 and 100 days of age were fed ad lib with a standard rat diet, and received ad lib tap water for drinking. To obtain time-pregnant females, the female rats in proestrus were pair-mated with male rats. The sperm-positive (day 0) rats were monitored for diestrus and body weight. On days 8 through 14 of gestation (3), the females received daily intraperitoneal injections of vinclozolin (100 mg/kg BW/day), or DDT (25 mg/kg BW/day) or dimethyl sulfoxide (DMSO) in oil (vehicle). The vinclozolin and DDT were obtained from Chem Service Inc. (West Chester, PA), and were injected in a 20 microliter DMSO/oil vehicle, as previously described (4). Treatment lineages are designated 'control' or 'vinclozolin' or 'DDT' lineages. The gestating female rats treated were designated as the F0 generation. The offspring of the F0 generation rats were the F1 generation. Non-littermate females and males aged 70-90 days from F1 generation of control or exposure lineages were bred to obtain F2 generation offspring within the lineage. The F2 generation rats were bred to obtain F3 generation offspring within the lineage. Individuals were maintained for 120 days and euthanized for sperm collection. At 120 days of age minimal disease is present, so the age was selected to avoid in confounding co-morbidities and disease artifacts. The F1- F3 generation offspring were not treated directly with DDT or vinclozolin. The control and exposure lineages were housed in the same room and racks with the same lighting, food and water, as previously described (4-6). All experimental protocols for the procedures with rats were pre-

approved by the Washington State University Animal Care and Use Committee (IACUC approval # 02568-39).

Epididymal sperm collection and DNA and RNA isolation

As previously described (1, 2), the epididymis was dissected free of connective tissue, a small cut made to the cauda and tissue placed in 5 ml of 1X PBS (phosphate buffer saline) for 10 minutes at 37°C and then kept at 4ºC to immobilize the sperm. The cauda epididymal tissue was minced, and the released sperm was centrifuged at 13, 000 x *g* and the pellet was stored at -20ºC until processed further. The sample was resuspended and sonicated to destroy any contaminating somatic cells and acrosome. This removed any somatic cell contamination due to the sonication resistance of the sperm head nuclei (7). The pellet was resuspended in NIM (Nucleus Isolation Medium) buffer (8), and then one hundred μl of sperm suspension was combined with 820 μL DNA extraction buffer and 80 μl 0.1M DTT (Dithiothreitol, Fisher Scientific, Cat # BP172-5, Lot #142653). The sample was incubated at 65°C for 15 minutes. Following this incubation, 80 μl proteinase K (20 mg/ml) was added and the sample was incubated at 55°C for at least 2 hours under constant rotation. Then, 300 μl of protein precipitation solution (Promega Genomic DNA Purification Kit, A795A) was added, and the sample was mixed thoroughly and incubated for 15 minutes on ice. The sample was centrifuged at 13,500 x *g* for 20 minutes at 4°C. One ml of the supernatant was transferred to a 2 ml tube and 2 μl of GlycoBlue and 1 ml of cold 100 % isopropanol were added. The sample was mixed well by inverting the tube several times then left in -20°C freezer for at least one hour. After precipitation, the sample was centrifuged at 12,500 x *g* for 20 minutes at 4°C. The

supernatant was taken off and discarded without disturbing the pellet. The pellet was washed with 70% cold ethanol by adding 500 μl of 70% ethanol to the pellet, and the tube was returned to the freezer for 20 minutes. After the incubation, the tube was centrifuged for 10 minutes at 4°C at 12,500 x *g* and the supernatant was discarded. The tube was spun again briefly to collect residual ethanol to the bottom of tube, and then as much liquid as possible was removed with a gel loading tip. The pellet was air-dried at RT until it looked dry (about 5 minutes). The pellet was then resuspended in 100 μl of nuclease free water.

Equal amounts of DNA or ncRNA from each individual's sperm from 3-4 different individuals for the F1 and F2 generations and 4-6 different individuals for the F3 generation were pooled and three different pools generation for each F1, F2, and F3 generation control and exposure lineage males. Therefore, each pool had different individuals, and there were three pools for each group. Two analyses of the same pool (n=10-17) were taken, one for DNA and one for ncRNA. Therefore, between 10 and 17 individuals were present in the three pools for the subsequent analysis.

RNA isolation

As previously described (1, 2), the F1-F3 generation exposure and control lineage male epididymal sperm were collected, processed, and stored at -80ºC until use (9). The total RNA (messenger RNA; long, noncoding RNA; ribosomal RNA; transfer RNA; sRNA) was isolated using the mirVana miRNA Isolation Kit (Life Technologies) following the manufacturer's instructions with modifications at the lysis stage. In brief, after addition of lysis buffer, the sperm pellets were manually homogenized, followed by a twenty-minute

incubation at 65°C. Samples were then placed on ice, and the default protocol was resumed. For quality control, RNA integrity numbers (RIN) were obtained by RNA 6000 Pico chips run on an Agilent 2100 Bioanalyzer (Agilent). A RIN of 2 – 4 indicates good sperm RNA quality. Concentration was determined using the Qubit RNA HS Assay Kit (ThermoFisher). Biological replicates of sperm were pooled by equal RNA content, and were concentrated using Agencourt AMPure XP beads (Beckman Coulter). Some pools had underrepresented replicates due to low concentration. In this case, if the Agilent profile was normal, the maximum RNA content from the replicate was used in the pool then the pool was concentrated. Abnormal Agilent profiles excluded the following samples from the pools: F1 DDT pool 2, sample 2; F2 DDT pool 1, samples 3 and 4. The pools and samples that were underrepresented are as follows: F1 control pool 3, samples 1 and 2; F2 control pool 2, sample 3; F2 DDT pool 1, samples 1 and 2; and F2 DDT pool 2, samples 1 and 5. Equal amounts of each pool were used in the final analysis.

Methylated DNA Immunoprecipitation MeDIP

As previously described (1, 2), methylated DNA Immunoprecipitation (MeDIP) with genomic DNA was performed as follows: rat sperm DNA pools were generated using the appropriate amount of genomic DNA from each individual for 3 pools each of control and exposure lineage animals. Genomic DNA was sonicated using the Covaris M220 the following way: the pooled genomic DNA was diluted to 130 µl with 1X TE into the appropriate Covaris tube. Covaris was set to 300 bp program, and the program was run for each tube in the experiment. 10 µl of each sonicated DNA was run on 1.5% agarose

gel to verify fragment size. The sonicated DNA was transferred from the Covaris tube to a 1.7 ml microfuge tube and the volume was measured. The sonicated DNA was then diluted with 1X TE buffer (10mM Tris HCl, pH7.5; 1mM EDTA) to 400 µl, heat-denatured for 10 minutes at 95˚C, then immediately cooled on ice for 10 minutes. Then 100µl of 5X IP buffer and 5µg of antibody (monoclonal mouse anti 5-methyl cytidine; Diagenode #C15200006) were added to the denatured sonicated DNA. The DNA-antibody mixture was incubated overnight on a rotator at 4˚C.

The following day, magnetic beads (Dynabeads M-280 Sheep anti-Mouse IgG; 11201D) were pre-washed as follows: The beads were resuspended in the vial, then the appropriate volume (50 µl per sample) was transferred to a microfuge tube. The same volume of Washing Buffer (at least 1 mL) was added and the bead sample was resuspended. Tube was then placed into a magnetic rack for 1-2 minutes and the supernatant was discarded. The tube was removed from the magnetic rack, and the beads were washed once. The washed beads were resuspended in the same volume of 1X IP buffer as the initial volume of beads. 50 µl of beads were added to the 500 µl of DNA-antibody mixture from the overnight incubation, then incubated for 2 hours on a rotator at 4˚C.

After the incubation, the bead-antibody-DNA complex was washed three times with 1X IP buffer as follows: The tube was placed into magnetic rack for 1-2 minutes and the supernatant was discarded, then it was washed with 1X IP buffer 3 times. The washed bead-DNA solution was then resuspended in 250 µl digestion buffer with 3.5 µl Proteinase K (20mg/ml). The sample was then incubated for 2-3 hours on a rotator at 55˚C, and 250 µl of buffered Phenol-Chloroform-Isoamylalcohol solution was added to the supernatant.

The tube was vortexed for 30 seconds then centrifuged at 12,500 x *g* for 5 minutes at room temperature. The aqueous supernatant was carefully removed and transferred to a fresh microfuge tube. 250µl chloroform was added to the supernatant from the previous step, vortexed for 30 seconds, and centrifuged at 12,500 x *g* for 5 minutes at room temperature. The aqueous supernatant was removed and transferred to a fresh microfuge tube. 2 µl of GlycoBlue (20mg/ml), 20 µl of 5M NaCl and 500 µl ethanol were added to the supernatant and mixed well, then precipitated in -20˚C freezer for 1 hour to overnight.

The precipitate was centrifuged at 12,500 x *g* for 20 minutes at 4˚C, and the supernatant was removed while not disturbing the pellet. The pellet was washed with 500 µl cold 70% ethanol in -20˚C freezer for 15 minutes then centrifuged again at 12,500 x *g* for 5 minutes at 4˚C, and the supernatant was discarded. The tube was spun again briefly to collect residual ethanol to bottom of tube, and as much liquid as possible was removed with gel loading tip. Pellet was air-dried at RT until it looked dry (about 5 minutes) then resuspended in 20 µl H_2O or 1X TE. DNA concentration was measured in Qubit (Life Technologies) with ssDNA kit (Molecular Probes Q10212).

ncRNA Sequencing Analysis

As previously described (1, 2), total RNA was used to construct large mRNA and ncRNA libraries for each pool. Libraries were constructed using the KAPA Stranded RNA-seq Library Preparation kit with RiboErase, according to the manufacturer's instructions, with some modifications. The adaptor and barcodes used were from NEBNext Multiplex Oligos for Illumina. Prior to PCR amplification, libraries were incubated at 37°C for 15 minutes with the USER enzyme (NEB). PCR cycle number was determined using qPCR

with the KAPA RealTime Library Amplification kit before final amplification. Size selection (300-700 bp) was performed using Agencourt AMPure XP beads (Beckman Coulter). Quality control was performed using Agilent DNA High Sensitivity chips (Agilent) and Qubit dsDNA high sensitivity assay (ThermoFisher). Libraries were pooled and loaded onto an Illumina NextSeq High Output v2 1x75 chip, and sequenced on an Illumina NextSeq 500 sequencer. Bioinformatics analysis was used to separate mRNA libraries from ncRNA libraries (see ncRNA bioinformatics section).

Prior to small library preparation, pooled total sperm RNA samples were enriched for small RNAs using the supplemental protocol for miRNA enrichment with SPRIselect by Beckman Coulter. Small RNA-enriched samples were used for small RNA library preparation, using the NEBNext Multiplex Small RNA Library Prep Set for Illumina, and barcoded with NEBNext Multiplex Oligos for Illumina. Size selection (135-170 bp) was performed using the Pippin Prep (Sage Science). Quality control was performed using Agilent DNA High Sensitivity chips (Agilent) and Qubit dsDNA high sensitivity assay (ThermoFisher). Libraries were pooled and loaded onto an Illumina NextSeq High Output v2 1x75 chip, and sequenced on an Illumina NextSeq 500 sequencer.

Histone Chromatin Immunoprecipitation ChIP-Seq -

As previously described (1, 2), histone chromatin immunoprecipitation with genomic DNA was performed as follows: rat sperm pools were generated using a total of 8 million sperm for 3 pools of control, DDT, and vinclozolin lineage animals. The control pools contained 5-6 individuals for a total of n=17 rats, the DDT pools contained 4 individuals for a total of n=12 rats per exposure group. Sperm from each animal was

sonicated for 10 seconds using a Fisher Sonic Dismembrator Model 300 then counted individually on a Neubaeuer improved cell prior to pooling. The sperm pools were filled up to 1 ml with 1X PBS. To reduce disulfide bonds, 50 µl of 1M DTT was added to each pool and incubated for 2 hours at room temperature under constant rotation. To quench any residual DTT in the reaction, 120 µl of 1M of NEM (N-Ethylmaleimide, Thermo Scientific, Prod # 23030, Lot # QB212210) was then added and incubated for 30 minutes at room temperature under constant rotation. The sperm cells were pelleted at 2,000 x *g* for 5 minutes at room temperature and the supernatant was discarded. Pellets were resuspended in 1X PBS. The mixture was spun again at 2,000 x *g* for 5 minutes at room temperature, and the supernatant was discarded.

The sperm cells were then resuspended in “complete buffer” in a ratio of 2 million sperm cells in 50 µl (as described (10)). “Complete buffer” was supplemented with tergitol 0.5% and DOC (sodium deoxycholate, Sigma Aldrich 30970). 50 µl of this mix was added to each aliquot. The tubes were homogenized and incubated for 20 minutes on ice. 10 Kuntz units of MNase (Roche, cat. no. 10107921001) were added and the samples incubated for 5 minutes at 37ºC. The reaction was stopped by the addition of 2 µl of EDTA (ethylenediaminetetraacetic acid, Sigma Aldrich, Batch # 077K0131) 0.5 M.

For the DDT samples, an enzymatic fragmentation of the chromatin was used. The sperm cells were then resuspended in “buffer 1” in a ratio of 2 million sperm cells per 50 µl (as described (10, 11)). “Complete buffer” was “buffer 1” supplemented with 0.5% tergitol (vol/vol) and 1% DOC (wt/vol) (sodium deoxycholate, Sigma Aldrich 30970). 50 µl of this supplemented buffer was added to each aliquot. The samples were mixed and incubated for 20 min on ice. 10 Kuntz units of MNase (NEB, M0247, Ispwich, MA) were added to

each sample and the samples incubated for 5 min at 37 °C. The reaction was stopped by the addition of 2 µl of 0.5 M EDTA.

For the vinclozolin samples, a sonication method was used to fragment the chromatin. Sperm cell DNA was divided into aliquots of 4 µg of DNA. These aliquots were sonicated using the Covaris M220 the following way: 4 µg of genomic DNA was resuspended in 130 µl of complete buffer supplemented with tergitol 0.5% and DOC 1%. Covaris was set to a 10 min "Chromatin shearing" program and the program was run for each tube in the experiment.

Ten µl of each sample was run on a 1.5% agarose gel to verify fragment size. The aliquots from the same sample were pooled back together and centrifuged at 12,500 x *g* for 10 minutes at room temperature. The supernatant was transferred to a fresh microfuge tube. 65 µl of protease inhibitors were added in each sample. 3 µl of antibody (monoclonal rabbit anti-histone H3, Millipore Sigma 05-928). The DNA-antibody mixture was incubated overnight on a rotator at 4ºC. The following day, magnetic beads (ChIP-Grade protein G magnetic beads, Cell Signaling 9006) were pre-washed as follows: the beads were resuspended in the vial, then the approximate volume (30 µl per sample) was transferred to a microfuge tube. The same volume of Washing Buffer (1X PBS with 0.1 % BSA and 2 mM EDTA) (at least 1 ml) was added and the bead sample was resuspended. Tube was then placed into a magnetic rack for 1-2 minutes, and the supernatant was discarded. The tube was removed from the magnetic rack and the beads were washed once. The washed beads were resuspended in the same volume of 1X IP buffer as the initial volume of beads. 30 µl of beads were added to the DNA-antibody mixture from the overnight incubation, then incubated for 2 hours on a rotator at 4ºC. After the incubation, the beads-

antibody-DNA complex was washed three times with 1X IP buffer as follows: the tube was placed into a magnetic rack for 1-2 minutes and the supernatant was discarded, then washed with 1X IP buffer 3 times. The washed beads-DNA solution was then resuspended in 300 μl of digestion buffer and 3 μl proteinase K (20 mg/ml). The sample was then incubated for 3 hours on a rotator at 56ºC. Then 300 μl of buffered Phenol-Chloroform-Isoamylalcohol solution was added to the supernatant, and the tube vortexed for 30 seconds then centrifuged at 12,500 x *g* for 10 minutes at room temperature. The aqueous supernatant was carefully removed and transferred to a fresh microfuge tube. Then 2 μl of GlycoBlue (20 mg/ml), a one-tenth volume of 3M sodium acetate and two volumes of ethanol were added. The mixture was vortexed 30 seconds then stored overnight in a -20ºC freezer.

The precipitate was centrifuged at 12,500 x *g* for 30 minutes at 4ºC, and the supernatant was removed while not disturbing the pellet. The pellet was washed with 500 μl cold 70% ethanol, then centrifuged again at 12,500 x *g* for 10 minutes at 4ºC and the supernatant was discarded. The tube was spun briefly to collect residual ethanol to the bottom of the tube, and as much liquid as possible was removed with gel loading tip. Pellet was air-dried at RT until it looked dry (about 5 minutes), then it was resuspended in 20 μl H_20. DNA concentration was measured in Qubit (Life Technologies) with brDNA kit (Molecular Probes Q32853).

MeDIP-Seq Analysis

As previously described (1, 2), the MeDIP pools were used to create libraries for next generation sequencing (NGS) using the NEBNext Ultra RNA Library Prep Kit for Illumina (NEB, San Diego, CA) starting at step 1.4 of the manufacturer's protocol to generate double stranded DNA. After this step the manufacturer's protocol was followed. Each pool received a separate index primer. NGS was performed at WSU Spokane Genomics Core using the Illumina HiSeq 2500 with a PE50 application, with a read size of approximately 50 bp and approximately 100 million reads per pool. Two to three libraries were run in one lane. The quality control of the sequencing libraries used FastQ and Trimmomatic. The sequence alignment was greater than 90% for all analyses.

Histone ChIP-Seq Analysis

As previously described (1, 2), the ChIP pools were used to create libraries for next generation sequencing (NGS) using the NEBNext Ultra II DNA Library Prep Kit for Illumina (NEB, San Diego, CA). The manufacturer protocol was followed. Each pool received a separate index primer. NGS was performed at WSU Spokane Genomics Core using Ilumina HiSeq 2500 with a PE50 application, with a read size of approximately 50 bp and approximately 35 million reads per pool. Six libraries were run in one lane.

Statistics and Bioinformatics

As previously described (1, 2), the basic read quality was verified using summaries produced by the FastQC program http://www.bioinformatics.babraham.ac.uk/projects/fastqc/. The raw reads were trimmed and filtered using Trimmomatic (12). The reads for each MeDIP and ChIP sample were

mapped to the Rnor 6.0 rat genome using Bowtie2 (13) with default parameter options. The mapped read files were then converted to sorted BAM files using SAMtools (14). To identify DMRs and DHRs, the reference genome was broken into 100 bp windows. Genomic windows with less than 40 mapped reads summed across all samples were removed prior to further analysis. The MEDIPS R package (15) was then used to calculate differential coverage between control and exposure sample groups. The edgeR p-value (16) was used to determine the relative difference between the two groups for each genomic window. Windows with an edgeR p-value less than 10^{-6} were considered DMRs or DHRs. The DMR/DHR edges were extended until no genomic window with a p-value less than 0.1 remained within 1000 bp of the DMR/DHR. CpG density and other information was then calculated for the DMR/DHR based on the reference genome.

DMRs and DHRs were annotated using the biomaRt R package (17) to access the Ensembl database (18). The genes that overlapped with DMR or DHR (within 10 kb) were then input into the KEGG pathway search (19, 20) to identify associated pathways. The DMR and DHR associated genes were then sorted into functional groups by consulting information provided by the DAVID (21), Panther (22), and Uniprot databases incorporated into an internal curated database (www.skinner.wsu.edu under genomic data). All molecular data has been deposited into the public database at NCBI [GEO # GSE109775 and GSE106125, NCIB SRA accession numbers: PRJNA430483 largeRNA (control and DTT), PRJNA430740 smallRNA (control, vinclozolin and DTT)]. The specific scripts used to perform the analysis can be accessed at github.com/skinnerlab and at www.skinner.wsu.edu/genomic-data-and-r-code-files.

A permutation analysis was used to demonstrate the epimutation overlaps were not random for data in Figs. 3 and 6 for the F1, F2 and F3 generation DMR overlaps and the F3 generation DMR, ncRNA and DHR overlaps a $p<0.001$ was obtained. A null model with epimutations randomly placed on the genome was used for the permutation analysis of overlapping sites. For each permutation, the epimutation sites were moved to new positions randomly in the genome. The minimum p-value for the moved epimutation at the new position was determined using the original analysis. If this p-value was less than or equal to 0.05, the site was assumed to be overlapping. The number of these overlaps across all sites was determined for every permutation. 1000 permutations were performed for each overlap. The p-value was then calculated as the proportion of permutations with overlaps equal or greater to the number obtained in the original analysis.

ncRNA Bioinformatics

As previously described (1, 2), the small ncRNA data was annotated as follows: low quality reads and reads shorter than 15nt were discarded by Cutadapt (23). The remaining reads were matched to known rat sncRNA, consisting of mature miRNA (miRBase, release 21), precursor miRNA (miRBase, release 21), tRNA (Genomic tRNA Database, rn5), piRNA (piRBase), rRNA (ENSEMBL, release 76), and mitochondrial RNA (ENSEMBL, release 76) using AASRA pipeline with default parameters (24). Read counts generated by AASRA was statistically normalized by DESeq2 (25).

The long ncRNA data was annotated as follows: The FASTX-Toolkit was used to remove adaptor sequences and the low quality reads from the RNA sequencing data of the mRNA libraries (12). To identify all the transcripts, we used Tophat2 and Cufflinks to

assemble the sequencing reads based on the Ensembl_Rnor_6.0 (26). The differential expression analyses were performed by Cuffdiff. The coding and the noncoding genes were primarily annotated through rat CDS data ensembl_Rnor_6.0. The non-annotated genes were extracted through our in-house script, then analyzed by CPAT, indicating the true non-coding RNAs (27).

References

1. Ben Maamar M, Sadler-Riggleman I, Beck D, McBirney M, Nilsson E, Klukovich R, et al. Alterations in sperm DNA methylation, non-coding RNA expression, and histone retention mediate vinclozolin-induced epigenetic transgenerational inheritance of disease. Environmental Epigenetics. 2018;4(2):1-19, dvy010.
2. Skinner MK, Ben Maamar M, Sadler-Riggleman I, Beck D, Nilsson E, McBirney M, et al. Alterations in sperm DNA methylation, non-coding RNA and histone retention associate with DDT-induced epigenetic transgenerational inheritance of disease. Epigenetics & Chromatin 2018;11(1):8, 1-24.
3. Nilsson EE, Anway MD, Stanfield J, Skinner MK. Transgenerational epigenetic effects of the endocrine disruptor vinclozolin on pregnancies and female adult onset disease. Reproduction. 2008;135(5):713-21.
4. Manikkam M, Guerrero-Bosagna C, Tracey R, Haque MM, Skinner MK. Transgenerational actions of environmental compounds on reproductive disease and identification of epigenetic biomarkers of ancestral exposures. PloS one. 2012;7(2):1-12, e31901.
5. Skinner MK, Manikkam M, Guerrero-Bosagna C. Epigenetic transgenerational actions of environmental factors in disease etiology. Trends Endocrinol Metab. 2010;21(4):214-22.
6. Anway MD, Leathers C, Skinner MK. Endocrine disruptor vinclozolin induced epigenetic transgenerational adult-onset disease. Endocrinology. 2006;147(12):5515-23.

7. Huang TT, Jr., Yanagimachi R. Inner acrosomal membrane of mammalian spermatozoa: its properties and possible functions in fertilization. Am J Anat. 1985;174(3):249-68.

8. Tateno H, Kimura Y, Yanagimachi R. Sonication per se is not as deleterious to sperm chromosomes as previously inferred. Biology of reproduction. 2000;63(1):341-6.

9. Wan LB, Bartolomei MS. Regulation of imprinting in clusters: noncoding RNAs versus insulators. Advances in genetics. 2008;61:207-23.

10. Hisano M, Erkek S, Dessus-Babus S, Ramos L, Stadler MB, Peters AH. Genome-wide chromatin analysis in mature mouse and human spermatozoa. Nature protocols. 2013;8(12):2449-70.

11. Ben Maamar M, Sadler-Riggleman I, Beck D, Skinner MK. Epigenetic Transgenerational Inheritance of Altered Sperm Histone Retention Sites. Scientific reports. 2018(8):5308, 1-10.

12. Bolger AM, Lohse M, Usadel B. Trimmomatic: a flexible trimmer for Illumina sequence data. Bioinformatics. 2014;30(15):2114-20.

13. Langmead B, Salzberg SL. Fast gapped-read alignment with Bowtie 2. Nature methods. 2012;9(4):357-9.

14. Li H, Handsaker B, Wysoker A, Fennell T, Ruan J, Homer N, et al. The Sequence Alignment/Map format and SAMtools. Bioinformatics. 2009;25(16):2078-9.

15. Lienhard M, Grimm C, Morkel M, Herwig R, Chavez L. MEDIPS: genome-wide differential coverage analysis of sequencing data derived from DNA enrichment experiments. Bioinformatics. 2014;30(2):284-6.

16. Robinson MD, McCarthy DJ, Smyth GK. edgeR: a Bioconductor package for differential expression analysis of digital gene expression data. Bioinformatics. 2010;26(1):139-40.
17. Durinck S, Spellman PT, Birney E, Huber W. Mapping identifiers for the integration of genomic datasets with the R/Bioconductor package biomaRt. Nature protocols. 2009;4(8):1184-91.
18. Cunningham F, Amode MR, Barrell D, Beal K, Billis K, Brent S, et al. Ensembl 2015. Nucleic acids research. 2015;43(Database issue):D662-9.
19. Kanehisa M, Goto S. KEGG: kyoto encyclopedia of genes and genomes. Nucleic acids research. 2000;28(1):27-30.
20. Kanehisa M, Goto S, Sato Y, Kawashima M, Furumichi M, Tanabe M. Data, information, knowledge and principle: back to metabolism in KEGG. Nucleic acids research. 2014;42(Database issue):D199-205.
21. Huang da W, Sherman BT, Lempicki RA. Systematic and integrative analysis of large gene lists using DAVID bioinformatics resources. Nature protocols. 2009;4(1):44-57.
22. Mi H, Muruganujan A, Casagrande JT, Thomas PD. Large-scale gene function analysis with the PANTHER classification system. Nature protocols. 2013;8(8):1551-66.
23. Martin M. Cutadapt removes adapter sequences from high-throughput sequencing reads. EMBnetjournal. 2011;17(1):10-2.
24. Tang C, Xie Y, Yan W. AASRA: An Anchor Alignment-Based Small RNA Annotation Pipeline. bioRxiv. 2017:132928.

25. Love MI, Huber W, Anders S. Moderated estimation of fold change and dispersion for RNA-seq data with DESeq2. Genome biology. 2014;15(12):550.

26. Trapnell C, Roberts A, Goff L, Pertea G, Kim D, Kelley DR, et al. Differential gene and transcript expression analysis of RNA-seq experiments with TopHat and Cufflinks. Nature protocols. 2012;7(3):562-78.

27. Wang L, Park HJ, Dasari S, Wang S, Kocher JP, Li W. CPAT: Coding-Potential Assessment Tool using an alignment-free logistic regression model. Nucleic acids research. 2013;41(6):e74.

Supplemental Figure and Table Legends

Supplemental Figure S1. Vinclozolin lineage F3 generation conserved DMR in common with DHR and F1 generation ncRNA. DMR gene associations with various pathology links. Legend for gene function shape in Supplemental Figure S2.

Supplemental Figure S2. DDT lineage F3 generation conserved DMR in common with DHR and F1 generation ncRNA. DMR gene associations with various pathology links. Legend insert for gene function shape.

Supplemental Table S1. Vinclozolin lineage F1 generation ncRNA & F1, F2 and F3 generation DMR overlap list. **(A)** F1 generation ncRNAs ($p<1e-04$) and F1 generation DMR ($p<0.05$). **(B)** F1 generation ncRNA ($p<1e-04$) and F2 generation DMR ($p<0.05$). **(C)** F1 generation ncRNA ($p<1e-04$) and F3 generation DMR ($p<0.05$).

Supplemental Table S2. DDT lineage F1 generation ncRNA & F1, F2 and F3 generation DMR overlap list. **(A)** F1 generation ncRNAs ($p<1e-04$) and F1 generation DMR ($p<0.05$). **(B)** F1 generation ncRNA ($p<1e-04$) and F2 generation DMR ($p<0.05$). **(C)** F1 generation ncRNA ($p<1e-04$) and F3 generation DMR ($p<0.05$).

Supplemental Table S3. F3 generation vinclozolin DMR & F3 generation DHR overlap list. F3 generation DMRs ($p<1e-06$) overlapping with F3 generation DHRs ($p<0.05$).

Supplemental Table S4. F3 generation DDT DMR & F3 generation DHR overlap list. F3 generation DMRs ($p<1e-06$) overlapping with F3 generation DHRs ($p<0.05$).

Supplemental Table S5. Vinclozolin lineage F1, F2, F3 generation DMR overlap list. F1 generation DMRs ($p<1e-06$) overlaps with F2 generation DMRs ($p<0.05$) and F3 generation DMRs ($p<0.05$).

Supplemental Table S6. DDT lineage F1, F2, F3 generation DMR overlap list. F1 generation DMRs ($p<1e-06$) overlaps with F2 generation DMRs ($p<0.05$) and F3 generation DMRs ($p<0.05$).
